# Supplementary material for: From sequence to enzyme mechanism using multi-label machine learning
Source: BMC Bioinformatics. 2014 May 19;15:150. doi: 10.1186/1471-2105-15-150 (PMC4229970; doi:10.1186/1471-2105-15-150)
Supplement: Additional file 2 — Java code of ml2db. Additional file ml2db_code.tar.gz contains the Java source code to run the multi-label machine learning experiments and save the results to database. The code’s Javadoc is included. [file 1471-2105-15-150-S2.zip › additional file 2/ml2db/ml2db/doc/test/dataharness/package-tree.html]

test.dataharness Class Hierarchy


---


|  |  |  |
| --- | --- | --- |
| **Overview** \| **Package** \| Class \| Use \| **Tree** \| **Deprecated** \| **Index** \| **Help** | |  |
| **PREV**   **NEXT** | **FRAMES**    **NO FRAMES**     **All Classes** |  |


---


## Hierarchy For Package test.dataharness

**Package Hierarchies:**: All Packages

---

## Class Hierarchy

- java.lang.Object
  - junit.framework.Assert
    - junit.framework.TestCase (implements junit.framework.Test)
      - test.dataharness.**ArffPropsFilesTest**- test.dataharness.**ArffPropsOneTest**- test.dataharness.**ArffPropsQueriesOneTest**- test.dataharness.**ArffPropsQueriesTest**- test.dataharness.**ArffPropsQueriesTwoTest**- test.dataharness.**ArffPropsTwoTest**- test.dataharness.**CreateDataTable**
                    - test.dataharness.**DataTableOneTest**- test.dataharness.**DataTableThreeTest**- test.dataharness.**DataTableTwoTest**
                    - test.dataharness.**DatabaseTest**- test.dataharness.**TestProjectParametersTest**
    - test.dataharness.**DataOne**- test.dataharness.**DataTwo**- test.dataharness.**LoadFirstValidArff**- uk.ac.ed.inf.enzml.**ProjectParameters**
            - test.dataharness.**TestProjectParameters**

---


|  |  |  |
| --- | --- | --- |
| **Overview** \| **Package** \| Class \| Use \| **Tree** \| **Deprecated** \| **Index** \| **Help** | |  |
| **PREV**   **NEXT** | **FRAMES**    **NO FRAMES**     **All Classes** |  |


---
